# Infodemiological data concerning silicosis in the USA in the period 2004–2010 correlating with real-world statistical data

**DOI:** 10.1016/j.dib.2016.11.021

**Published:** 2016-11-13

**Authors:** Nicola Luigi Bragazzi, Guglielmo Dini, Alessandra Toletone, Francesco Brigo, Paolo Durando

**Affiliations:** a School of Public Health, Department of Health Sciences (DISSAL), University of Genoa, Via Antonio Pastore 1, Genoa 16132, Italy; b Department of Health Sciences, Postgraduate School in Occupational Medicine, University of Genoa and Occupational Medicine Unit, I.R.C.C.S. University Hospital San Martino - IST National Institute for Cancer Research, Genoa, Italy; c Department of Health Sciences, Postgraduate School in Occupational Medicine, University of Genoa, Genoa, Italy; d Department of Neurology, Franz Tappeiner Hospital, Merano, Italy; e Department of Neurological, Biomedical, and Movement Sciences, University of Verona, Italy; f Unità operativa Medicina del lavoro, IRCCS AOU San Martino-IST, Genoa, Italy

**Keywords:** Infodemiology and infoveillance, Internet, Occupational medicine and hygiene, Web 2.0, Work-related diseases

## Abstract

This article reports data concerning silicosis-related web-activities using Google Trends (GT) capturing the Internet behavior in the USA for the period 2004–2010. GT-generated data were then compared with the most recent available epidemiological data of silicosis mortality obtained from the Centers for Disease Control and Prevention for the same study period. Statistically significant correlations with epidemiological data of silicosis (*r*=0.805, *p*-value <0.05) and other related web searches were found. The temporal trend well correlated with the epidemiological data, as well as the geospatial distribution of the web-activities with the geographic epidemiology of silicosis.

**Specifications Table**

**Table:** 

Subject area	*Medicine*
More specific subject area	*Occupational medicine*
Type of data	*Figure, tables*
How data was acquired	*Outsourcing of Google Trends site and the Centers for Disease Control and Prevention (CDC) site*
Data format	*Raw, analyzed*
Experimental factors	*Google Trends search volumes were obtained through graphs and heat-maps*
Experimental features	*Validation of Google Trends-based data with “real-world” data taken from the CDC site was performed by means of correlational analysis*
Data source location	USA
Data accessibility	Data are within this article

**Value of the data**

• Google Trends (GT)-based data (*infodemiological* data) could be useful for scientific community, researchers and occupational physicians in that they show good correlation with “real world” data obtained from the Centers for Disease Control and Prevention site, thus proving to be reliable.
• These data could be further statistically processed, analyzed, refined and validated in such a way to complement traditional surveillance of silicosis, providing data quicker and in real time.
• These data could be used to understand occupational diseases-related web activities.
• To our knowledge, this is the first analysis of web search behavior related to an occupational disease, namely silicosis, carried out both in quantitative and qualitative terms.

## 1 Data

**Fig. 1:**
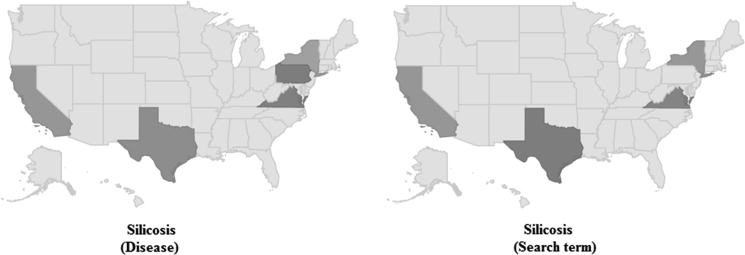
Google Trends-generated heat-map showing the regional interest for silicosis in the USA. In particular, it can be noticed that silicosis-related web searches are concentrated in some counties (namely, California, Texas, New York, Pennsylvania, and Virginia).

This article contains infodemiological data on silicosis searched in the USA in the study period 2004–2010, obtained from Google Trends (GT) (Fig. 1). These data well correlated with “real-world” data obtained from the Centers for Disease Control and Prevention (CDC) site for the same study period (Table 1, Table 2, Table 3).

## 2 Experimental design, materials and methods

GT (available at https://www.google.com/trends) was exploited in order to capture Internet activities and interest related to silicosis. GT was mined in the USA, looking for “silicosis” as keyword, and using both “search term” (data directly available at https://www.google.com/trends/explore?date=2004-01-01%202010-12-31&geo=US&q=Silicosis) and “search topic” [Disease] (data directly available at https://www.google.com/trends/explore?date=2004-01-01%202010-12-31&geo=US&q=%2Fm%2F02yw8n) as search strategy options, from 2004 to 2010. Data downloadable from GT are available as monthly data, in comma-separated values (CSV) format.

“Real-world” statistical data, both raw and adjusted, were collected from the CDC site for the same study period 2004–2010 [1], [2], [3], [4], [5].

Correlational analysis was carried out between the GT-based search volumes and the “real-world” statistical data about silicosis. A list of silicosis-related terms (clinical symptoms and other associated diseases) was further searched and their flux volumes were correlated with the silicosis hit-search data and the epidemiological data (namely, death rate and number of deaths).

All statistical analyses were carried out using the Statistical Package for Social Science version 23.0 (SPSS, IBM, IL, USA) and STATISTICA version 12 (StatSoft Inc., Tulsa, OK, USA). Figures with a *p*-value <0.05 were considered significant.

For further details, the reader is referred to [6].

## Figures and Tables

**Table 1:** Pearson׳s correlation between Google Trends-based data and epidemiological data in the study period 2004–2010.

**Variable**		**GT-based silicosis (Disease)**	**GT-based silicosis (search term)**
**Gender**			
			
Female	Correlation coefficient	−0.145	−0.144
	Significance level P	0.7562	0.7588
			
Male	Correlation coefficient	*0.778*⁎	*0.765*⁎
	Significance level *P*	*0.0394*	*0.0453*

**Ethnicities**			
			
**White**	Correlation coefficient	0.713	0.696
	Significance level *P*	0.0720	0.0825
			
Female	Correlation coefficient	0.010	−0.001
	Significance level *P*	0.9832	0.9980
			
Male	Correlation coefficient	*0.767*⁎	*0.755*⁎
	Significance level *P*	*0.0441*	*0.0498*
			
**Black**	Correlation coefficient	*0.841*⁎	*0.847*⁎
	Significance level *P*	*0.0177*	*0.0162*
			
Female	Correlation coefficient	*−0.176*	*−0.162*
	Significance level *P*	*0.7066*	*0.7281*
			
Male	Correlation coefficient	*0.855*⁎	*0.859*⁎
	Significance level *P*	*0.0143*	*0.0132*
			
**Other**	Correlation coefficient	−0.135	−0.162
	Significance level *P*	0.7731	0.7286
			
Female	Correlation coefficient	−0.292	−0.254
	Significance level *P*	0.5249	0.5833
			
Male	Correlation coefficient	−0.019	−0.055
	Significance level *P*	0.9676	0.9074

**Adjusted white**			
			
Female	Correlation coefficient	−0.015	−0.007
	Significance level *P*	0.9751	0.9876
			
Male	Correlation coefficient	*0.787*⁎	*0.778*⁎
	Significance level *P*	*0.0357*	*0.0396*

**Adjusted black**			
			
Female	Correlation coefficient	−0.155	−0.149
	Significance Level P	0.7396	0.7507
			
Male	Correlation coefficient	*0.864*⁎	*0.867*⁎
	Significance level *P*	*0.0122*	*0.0116*

**Adjusted other**			
			
Female	Correlation coefficient	−0.292	−0.254
	Significance level *P*	0.5249	0.5833
			
Male	Correlation coefficient	0.030	0.004
	Significance level *P*	0.9490	0.9939
			
**Adjusted overall**	Correlation coefficient	*0.823*⁎	*0.816*⁎
	Significance level *P*	*0.0231*	*0.0253*

**Age**			
			
age 15–24	Correlation coefficient	−0.070	−0.018
	Significance level *P*	0.8813	0.9695
			
age 25–34	Correlation coefficient	−0.657	−0.656
	Significance level *P*	0.1091	0.1092
			
age 35–44	Correlation coefficient	0.501	0.533
	Significance level *P*	0.2520	0.2179
			
age 45–54	Correlation coefficient	0.308	0.278
	Significance Level P	0.5017	0.5466
			
age 55–64	Correlation coefficient	0.457	0.468
	Significance level *P*	0.3031	0.2898
			
age 65–74	Correlation coefficient	0.619	0.622
	Significance level *P*	0.1379	0.1357
			
age 75–84	Correlation coefficient	0.701	0.677
	Significance level *P*	0.0792	0.0949
			
age 85-on	Correlation coefficient	0.462	0.442
	Significance level *P*	0.2966	0.3208
			
No. >45	Correlation coefficient	0.747	0.730
	Significance level *P*	0.0535	0.0623
			
No. 15–44	Correlation coefficient	0.291	0.334
	Significance level *P*	0.5262	0.4636
			
**Underlying**	Correlation coefficient	*−0.850*⁎	*−0.832*⁎
	Significance level *P*	*0.0154*	*0.0203*
			
**Number of deaths**	Correlation coefficient	*0.759*⁎	*0.746*
	Significance level *P*	*0.0477*	*0.0542*
			
**Death rate**	Correlation coefficient	*0.805*⁎	*0.794*⁎
	Significance level *P*	*0.0291*	*0.0329*

⁎ Statistically significant, with *p*-value <0.05.

**Table 2:** Pearson׳s correlation between GT-based data and clinical symptoms/diseases associated with silicosis.

**Variable**		**GT-based silicosis (Disease)**	**GT-based silicosis (search term)**
**Associated diseases**			
			
Lung cancer	Correlation coefficient	0.714	0.740
	Significance level *P*	0.0712	0.0574
			
Laryngeal cancer	Correlation coefficient	−0.749	−0.786
	Significance level *P*	0.0526	0.0360
			
Rheumatoid arthritis	Correlation coefficient	*0.793*⁎	*0.767*⁎
	Significance level *P*	*0.0333*	*0.0443*
			
Systemic Lupus Erythematosus	Correlation coefficient	*0.869*⁎	*0.865*⁎
	Significance level *P*	*0.0112*	*0.0120*
			
Scleroderma	Correlation coefficient	*0.918*⁎	*0.934*⁎
	Significance level *P*	*0.0035*	*0.0021*
			
Tubercolosis	Correlation coefficient	0.083	0.106
	Significance level *P*	0.8588	0.8217

**Symptoms**			
			
Anorexia	Correlation coefficient	0.220	0.184
	Significance level *P*	0.6348	0.6931
			
Cough	Correlation coefficient	−0.740	*−0.770*⁎
	Significance level *P*	0.0571	*0.0429*
			
Dyspnea	Correlation coefficient	−0.725	*−0.757*⁎
	Significance level *P*	0.0654	*0.0490*
			
Fatigue	Correlation coefficient	−0.576	−0.612
	Significance level *P*	0.1756	0.1438
			
Fever	Correlation coefficient	*−0.848*⁎	*−0.869*⁎
	Significance level *P*	*0.0158*	*0.0110*
			
Respiratory failure	Correlation coefficient	*−0.939*⁎⁎	*−0.939*⁎⁎
	Significance level *P*	*0.0017*	*0.0017*
			
Tachipnea	Correlation coefficient	*−0.937*⁎⁎	*−0.941*⁎⁎
	Significance level *P*	*0.0018*	*0.0016*

⁎ Statistically significant, with *p*-value <0.05;

⁎⁎ Statistically significant, with *p*-value <0.01.

**Table 3:** Pearson׳s correlation between GT-based data concerning clinical symptoms/diseases associated with silicosis and silicosis epidemiological data (namely, death rate and number of deaths) in the study period 2004–2010.

**Variable**		**GT-based silicosis (Disease)**	**GT-based silicosis (search term)**
**Associated diseases**			
			
Lung cancer	Correlation coefficient	0.736	0.697
	Significance level *P*	0.0595	0.0818
			
Laryngeal cancer	Correlation coefficient	−0.680	−0.628
	Significance level *P*	0.0929	0.1308
			
Rheumatoid arthritis	Correlation coefficient	0.476	0.445
	Significance level *P*	0.2797	0.3165
			
Systemic Lupus Erythematosus	Correlation coefficient	0.455	0.399
	Significance level *P*	0.3051	0.3755
			
Scleroderma	Correlation coefficient	*0.861*⁎	*0.823*⁎
	Significance level *P*	*0.0129*	*0.0230*
			
Tubercolosis	Correlation coefficient	−0.007	−0.030
	Significance level *P*	0.9879	0.9484

**Symptoms**			
			
Anorexia	Correlation coefficient	−0.161	−0.175
	Significance level *P*	0.7299	0.7080
			
Cough	Correlation coefficient	*−0.817*⁎	*−0.784*⁎
	Significance level *P*	*0.0247*	*0.0370*
			
Dyspnea	Correlation coefficient	*−0.790*⁎	*−0.754*
	Significance level *P*	*0.0347*	*0.0503*
			
Fatigue	Correlation coefficient	−0.753	−0.729
	Significance level *P*	0.0505	0.0632
			
Fever	Correlation coefficient	*−0.820*⁎	*−0.776*
	Significance level *P*	*0.0240*	*0.0401*
			
Respiratory failure	Correlation coefficient	*−0.864*⁎	*−0.825*⁎
	Significance level *P*	*0.0121*	*0.0225*
			
Tachipnea	Correlation coefficient	*−0.902*⁎⁎	*−0.867*⁎
	Significance level *P*	*0.0054*	*0.0115*

⁎ Statistically significant, with *p*-value <0.05;

⁎⁎ Statistically significant, with *p*-value <0.01.
